# Supplementary material for: Green Synthesis of Free Standing Cellulose/Graphene Oxide/Polyaniline Aerogel Electrode for High-Performance Flexible All-Solid-State Supercapacitors
Source: Nanomaterials (Basel). 2020 Aug 7;10(8):1546. doi: 10.3390/nano10081546 (PMC7466413; doi:10.3390/nano10081546)
Supplement: Supplementary file 1 [file nanomaterials-10-01546-s001.pdf]

Supplementary Materials

Green Synthesis of Free Standing Cellulose/Graphene Oxide/Polyaniline Aerogel Electrode for High-Performance Flexible All-Solid-State Supercapacitors

Yueqin Li [1,2,*], Zongbiao Xia [1,2], Qiang Gong [1,2], Xiaohui Liu [1,2], Yong Yang [1,2], Chen Chen [2] and Changhao Qian [2]

[1] Co-Innovation Center of Efficient Processing and Utilization of Forest Resources, Nanjing Forestry University, Nanjing 210037, China; zongbiaoxia@163.com (Z.X.); qianggong@163.com (Q.G.); xiaohuiliu@163.com (X.L.); yongyang@163.com (Y.Y.)

[2] College of Chemical Engineering, Jiangsu Key Lab for the Chemistry and Utilization of Agricultural and Forest Biomass, Nanjing Forestry University, Nanjing 210037, China; chenchen@163.com (C.C.); changhaoqian@163.com (C.Q.)

* Correspondence: yueqinli@njfu.edu.cn; Tel.: +86-025-85427024

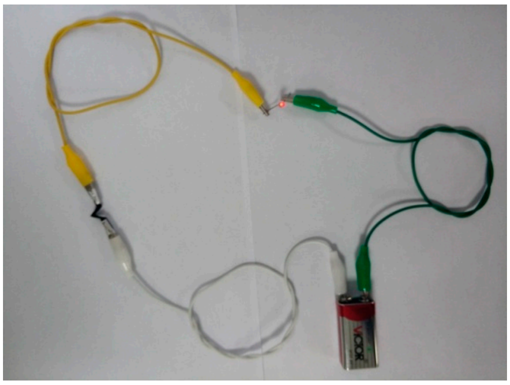

Figure S1. The cellulose/$GO_{3.5}$/PANI film was folded and used as a wire to light up a red LED.

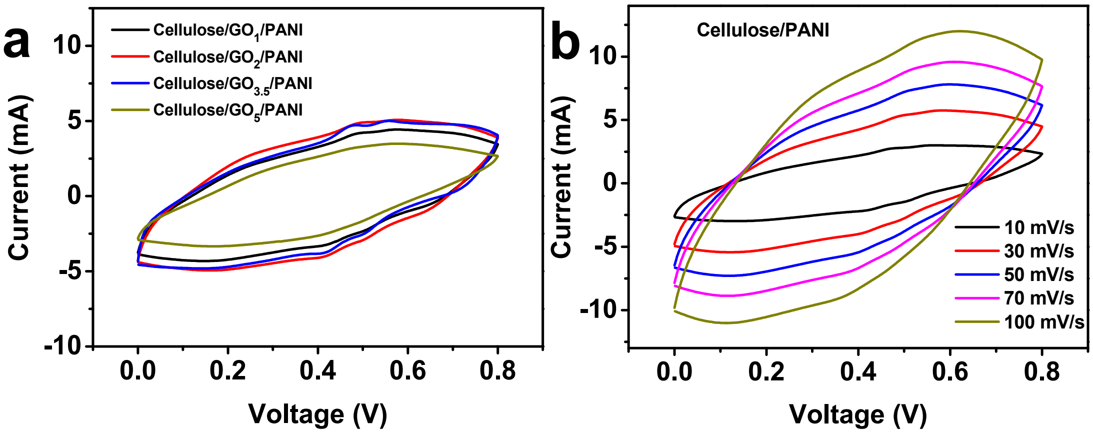

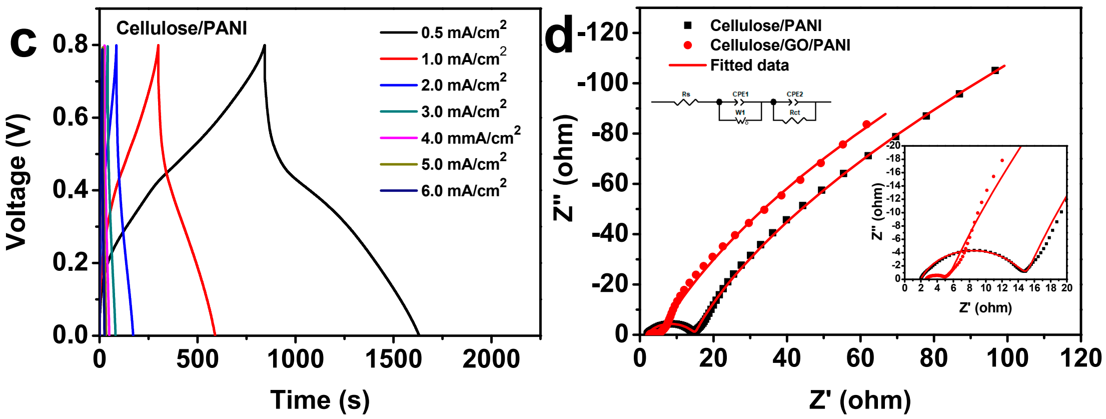

Figure S2. (**a**) CV curves of the cellulose/GO1.0/PANI, cellulose/GO2.0/PANI, cellulose/GO3.5/PANI and cellulose/GO5.0/PANI samples at 10 mV/s. (**b**) CV curves of cellulose/PANI sample. (**c**) GCD curves of cellulose/PANI sample. (**d**) Nyquist plot of the cellulose/PANI and cellulose/GO/PANI with an equivalent circuit in the inset.

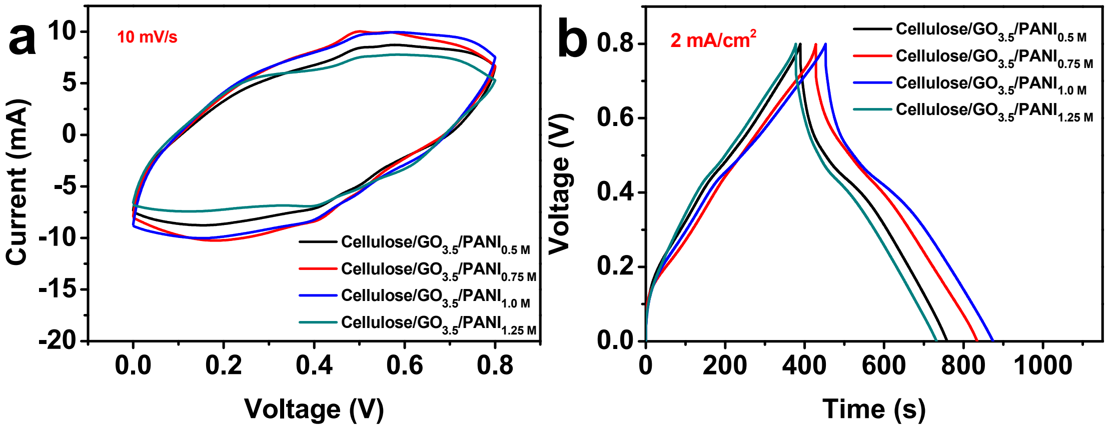

Figure S3. (**a**) CV curves of the cellulose/GO3.5/PANI0.5M, cellulose/GO3.5/PANI0.75M, cellulose/GO3.5/PANI1.0M (namely cellulose/GO3.5/PANI sample in the manuscript) and cellulose/GO3.5/PANI1.25M samples at 10 mV/s. (**b**) GCD curves of the cellulose/GO3.5/PANI0.5M, cellulose/GO3.5/PANI0.75M, cellulose/GO3.5/ PANI1.0M and cellulose/GO3.5/PANI1.25M samples.

Table S1. Comparison of electrochemical performance of various electrodes based on conducting fillers/cellulose composites.

Materials	Maximum Cs (mF/cm²)	Cyclic stability	Reference
Graphene/cellulose paper	81 (1 mV/s)	99.1% (5000)	[1]
SWCNT/PANI/cellulose	330 (0.2 mA/cm²)	79% (1000)	[2]
Graphite /PANI/paper	355.6 (0.5 mA/cm²)	-	[3]
GO/PPy	387.6 (0.2 mA/cm²)	84.8% (5000)	[4]
CNT/PANI hydrogel	680 (1 mA/cm²)		[5]
PANI/Graphite paper	176 (0.2 mA/cm²)	-	[6]
PANI/RGO film	718 (0.45 A/g)	74% (500)	[7]
Graphene/PANI/Graphene	190.6 (0.5 mA/cm²)	96% (1000)	[8]
RGO/PPy CCFs paper	363 (0.5 mA/cm²)	-	[9]
Carbon cloth-PANI-rGO	471 (0.5 mA/cm²)	75.5% (10000)	[10]
PANI/CNT/Graphene	465 (1 mA/cm²)	84% (1000)	[11]
PANI/GO/CNT	510.5 (1 A/g)	-	[12]
CNFs-RGO/PPy	334 (0.1 mA/cm²)	100% (2000)	[13]
Cellulose/GO3.5/PANI	1218 (1.0 mA/cm²)	83.5% (1000)	This work

References

1. Weng, Z.; Su, Y.; Wang, D.-W.; Li, F.; Du, J.; Cheng, H.-M. Graphene-cellulose paper flexible supercapacitors. *Adv. Energy Mater.* **2011**, *1*, 917–922.
2. Ge, D.; Yang, L.; Fan, L.; Zhang, C.; Xiao, X.; Gogotsi, Y.; Yang, S. Foldable supercapacitors from triple networks of macroporous cellulose fibers, single-walled carbon nanotubes and polyaniline nanoribbons. *Nano Energy* **2015**, *11*, 568–578.
3. Yao, B.; Yuan, L.; Xiao, X.; Zhang, J.; Qi, Y.; Zhou, J.; Zhou, J.; Hu, B.; Chen, W. Paper-based solid-state supercapacitors with pencil-drawing graphite/polyaniline networks hybrid electrodes. *Nano Energy* **2013**, *2*, 1071–1078.
4. Cao, J.; Wang, Y.; Chen, J.; Li, X.; Walsh, F.C.; Ouyang, J.-H.; Jia, D.; Zhou, Y. Three-dimensional graphene oxide/polypyrrole composite electrodes fabricated by one-step electrodeposition for high performance supercapacitors. *J. Mater. Chem. A* **2015**, *3*, 14445–14457.
5. Zeng, S.; Chen, H.; Cai, F.; Kang, Y.; Chen, M.; Li, Q. Electrochemical fabrication of carbon nanotube/polyaniline hydrogel film for all-solid-state flexible supercapacitor with high areal capacitance. *J. Mater. Chem. A* **2015**, *3*, 23864–23870.
6. Li, K.; Liu, X.; Chen, S.; Pan, W.; Zhang, J. A flexible solid-state supercapacitor based on graphene/polyaniline paper electrodes. *J. Energy Chem.* **2019**, *32*, 166–173.
7. Kim, M.; Lee, C.; Jang, J. Fabrication of highly flexible, scalable, and high-performance supercapacitors using polyaniline/reduced graphene oxide film with enhanced electrical conductivity and crystallinity, *Adv. Funct. Mater.* **2014**, *24*, 2489–2499.
8. Chi, K.; Zhang, Z.; Xi, J.; Huang, Y.; Xiao, F.; Wang, S.; Liu, Y. Freestanding graphene paper supported three-dimensional porous graphene-polyaniline nanocomposite synthesized by inkjet printing and in flexible all-solid-state supercapacitor. *Acs Appl Mater. Interfaces* **2014**, *6*, 16312–16319.
9. Lyu, S.; Chang, H.; Fu, F.; Hu, L.; Huang, J.; Wang, S. Cellulose-coupled graphene/polypyrrole composite electrodes containing conducting networks built by carbon fibers as wearable supercapacitors with excellent foldability and tailorability. *J. Power Sources* **2016**, *327*, 438–446.
10. Du, P.; Dong, Y.; Kang, H.; Yang, X.; Wang, Q.; Niu, J.; Wang, S.; Liu, P. Graphene-wrapped polyaniline nanowire array modified functionalized of carbon cloth for high-performance flexible solid-state supercapacitor. *ACS Sustain. Chem. Eng.* **2018**, *6*, 14723–14733.
11. Vedhanarayanan, B.; Huang, T.-H.; Lin, T.-W. Fabrication of 3D hierarchically structured carbon electrode for supercapacitors by carbonization of polyaniline/carbon nanotube/graphene composites. *Inorg. Chim. Acta* **2019**, *489*, 217–223.
12. Jiang, Q.; Shang, Y.; Sun, Y.; Yang, Y.; Hou, S.; Zhang, Y.; Xu, J.; Cao, A. Flexible and multi-form solid-state supercapacitors based on polyaniline/graphene oxide/CNT composite films and fibers. *Diam. Relat. Mater.* **2019**, *92*, 198–207.
13. Mo, M.; Chen, C.; Gao, H.; Chen, M.; Li, D. Wet-spinning assembly of cellulose nanofibers reinforced graphene/polypyrrole microfibers for high performance fiber-shaped supercapacitors. *Electrochim. Acta* **2018**, *269*, 11–20.
